# Torpedo Retinopathy or Chorioretinopathy?

**DOI:** 10.18502/jovr.v17i4.12345

**Published:** 2022-11-29

**Authors:** Mahmoud Dehghan, Ramin Nourinia, Sahba Fekri, Seyed-Hossein Abtahi

**Affiliations:** ^1^Ophthalmic Research Center, Research Institute for Ophthalmology and Vision Science, Shahid Beheshti University of Medical Sciences, Tehran, Iran; ^2^Department of Ophthalmology, Labbafinejad Medical Center, Shahid Beheshti University of Medical Sciences, Tehran, Iran; ^4^https://orcid.org/0000-0001-5102-1109; ^5^https://orcid.org/0000-0002-1459-6752

##  Dear Editor,

We read with great interest the recent case series by Venkatesh and colleagues about the spectrum and features of Torpedo lesions described in nine subjects.^[1]^ As far as we are aware,the *Journal of Ophthalmic and Vision Research * has recently published another report on the same topic,^[2]^ and we have encountered such rare cases in our daily practice at Labbafinejad Medical Center as one of the main tertiary centers for retinal diseases. Herein, we would like to draw the kind attention of readers toward some pathophysiologic issues about the origin of Torpedo lesions.

In 1992, Roseman and Gass^[3]^ described such lesions as solitary hypopigmented nevus of the retinal pigment epithelium (RPE) and defined them as rare congenital anomalies of the RPE that disrupt the outer retina. As well as the known term “Torpedo maculopathy (TM)”, other nomenclatures, are sparsely used as paramacular albinotic spot syndrome, congenital hypomelanotic freckle, and atypical macular coloboma.^[1,2,3,4]^

The embryonal origin of these congenital lesions is believed to be rooted in maldevelopment of RPE and disturbances of choroidal vasculature. Furthermore, as discussed in a very recent systematic review, subtypes II and III of Torpedo lesions have been suggested to be classified as choroidal cavitary disorders.^[1,2,4,5,6]^ In OCT angiography (OCTA) studies, the primary site of malformation was found to be in the RPE/choriocapillaris complex.^[6]^

Herein, we would like to underline the concluding statement of Venkatesh and colleagues,^[1]^ where they stated that Torpedo lesions can occur away from the macula with the same appearance of TM. They proposed a change in the nomenclature for torpedo lesions in the retina from “torpedo maculopathy” to “torpedo retinopathy”. Consistently, we endorse the notion that such lesions are not exclusively limited to the posterior pole and they can be seen at near-peripheral retina. Nevertheless, if we plan to revise the nomenclature, the choroidal origin of Torpedo lesions should also be addressed. In conclusion, we believe that *Torpedo chorioretinopathy* fits best as a comprehensive nomenclature covering both concerns about the pathophysiologic origin and the location of the Torpedo lesions.

##  Financial Support and Sponsorship

None.

##  Conflicts of Interest

None.
